# Multifractal evidence of nonlinear interactions stabilizing posture for phasmids in windy conditions: A reanalysis of insect postural-sway data

**DOI:** 10.1371/journal.pone.0202367

**Published:** 2018-08-23

**Authors:** Damian G. Kelty-Stephen

**Affiliations:** Department of Psychology, Grinnell College, Grinnell, Iowa, United States of America; University of Minnesota, UNITED STATES

## Abstract

The present work is a reanalysis of prior work documenting postural sway in phasmids (i.e., “stick insects”) [1]. The prior work pursued the possibility that postural sway was an evolutionary adaptation supporting motion camouflage to avoid the attention of predators. For instance, swaying along with leaves blown by the wind might reduce the likelihood of standing out to a predator. The present work addresses the alternative—but by no means conflicting and perhaps more explanatory—proposal that phasmid postural sway carries evidence of the tensegrity-like structures allowing postural stabilization under wind-like stimulation. Tensegrity structures are prestressed architectures embodying nonlinear interactions across scales of space and time that provide context-sensitive responses faster than neural tissue can support. Multifractal modeling of the postural-displacement series initially recorded in [1] offers a metric equally effective for quantifying complexity of phasmid postural sway under wind stimulation as for quantifying complexity of human postural sway [2–7]. Furthermore, multifractal modeling offers a means to demonstrate empirically the nonlinear interactions across space and time scales in body-wide coordination that tensegrity-based hypotheses predict. Specifically, multifractal modeling allows diagnosing the strength and direction of nonlinear interactions across time scale as the difference between multifractal estimates for the original postural-displacement series and for a sample of best-fitting linear models of the series. The reduction of postural sway directly following the application of wind stimulus appears as a significant decrease in the multifractal structure for original postural-displacement series as compared to best-fitting linear models of those series. This decrease indicates the capacity for nonlinear interactions across time scale to constrict variability, which is an aspect of nonlinear dynamics often overshadowed by the possibility that nonlinearity can produce more variability. This work offers the longer-range opportunity that multifractal modeling could provide a common language within which to coordinate behavioral sciences across a wide range of species.

## Introduction

Postural sway is pervasive feature of context-sensitive, goal-directed behaviors. It spans such a wide range of species that, at first glance, it seems that the research questions surrounding postural sway in one species have little to do with another. For instance, in a human model, postural sway raises questions about stability of upright standing and fall risk, one of the leading causes of death in the elderly [8]. In their study of phasmids (“stick insects”), Bian, Elgar, and Peters [1] used postural sway to open up a class of fascinating questions regarding motion camouflage, e.g., whether or not insects will sway to blend in with surrounding foliage. However, despite these disparate themes, postural sway may exhibit common threads across such disparate species, common threads that may inform questions about important features of context-sensitive goal-directed behavior generic to different species.

In the present work, I revisited evidence from postural sway in phasmid and attempts find a geometric parallel between phasmid postural sway and human postural sway. Far from attempting to make an abstract geometrical comparison interesting in its own right, I propose that this geometrical resemblance may point to deeper connections in terms of commonalities in how these different species make use of perceptual information. Much like the rest of human movement physiology from autonomic to voluntary processes [9–14], the geometry of human postural sway has appeared to be multifractal [2–7], and postural sway is a key source of perceptual information as an organism coordinates visual information, kinesthesis, and haptic information to support stable, context-sensitive behaviors [15–19]. The co-occurrence of multifractal structure and of coordination of perceptual information may be far from coincidence. On the contrary, a tensegrity hypothesis about full-bodied movement suggests that, across many grains of analysis and for a wide variety of species, the multifractal shape of movement variability is the key signature of a bodily architecture embodying a richly textured balancing of tension and compression elements from the cellular to the organismic scales [20]. I hoped here to demonstrate a potential use for empirical estimates of multifractal geometry in phasmids to understand how tensegrity like bodily coordinations serve to stabilize posture in an uncertain context.

### Tensegrity hypothesis: Body-wide coordination of movement with ultrafast responses

The tensegrity hypothesis originates from neither insect nor human behavior but comes from work originating in cell and tissue biology suggesting that various biochemical and physiological processes depend on a vast network balancing tension and compression elements built across many spatial scales. The term “tensegrity” is a portmanteau short for “tensional integrity.” This multiscale network encompasses actin-myosin chains at the cellular level, extends across the extracellular matrix (ECM), and continues up (in vertebrates) to the macroscopic scale of musculoskeletal system where muscles serve as tensional elements balanced with the compression elements provided by bone. At this musculoskeletal scale, the connective tissue analogue to extracellular matrix appears as the fascia, an extremely tensile material whose manifold deformations provide global, contextual support for the more focal events of action potentials and flexions of innervated muscle tissue. Tensegrity architecture supports “mechanotransduction,” the rapid propagation of local perturbations across the global architecture of an entire cell, tissue, or organ—at speeds beyond the limits of more local modes of transmission, such as second-messenger systems or neural transmission [21].

It is these “ultrafast” propagations of perturbation across vast distances within biological systems [22;23] have elsewhere prompted physiologists and movement scientists to coin the term “preflex” to indicate a rapid motoric response based sooner in mechanical tensions than in neural transmission [24;25]. That is, tensegrity architectures prompt responses sooner than a reflex can, and the global reach of the fascial/ECM serves to situate neural processes in the context and so to anticipate specifically those contextual demands for neural processes in the unfolding behaviors. Indeed, roboticists have already incorporated these insights about tensegrity and about preflexes as central themes for their projects to design artificially intelligent agents that will be anticipatory, context-sensitive in cluttered and uncertain environmental conditions [26]. Behavioral and psychological sciences have seen a much more gradual interest in tensegrity, with observations of ultrafast responses both in phasmids [27] (as well as other insects, e.g. wasps [28]) and in humans [3;29–32]) motivating the suspicion that tensegrity structures area relevant to behavior. But the appearance of the tensegrity hypothesis in new theorizing behavioral and psychological sciences has been far from central. It may seem strange this intriguing notion of tensegrity has motivate disparate rates of progress in different fields.

### Behavioral- and psychological-science receptivity to tensegrity hypotheses: Slower than ultrafast but potentially informed by multifractal geometry

Some of the difference in receptivity in different fields has much to do with the fields’ difference reliance (or not) on the freedom to build an autonomous system and, likewise, the fields’ different reliance (or not) on randomized controlled trials to adjudicate the identification of a cause. To some degree, it is no surprise that the appreciation for tensegrity structures in robotics has outpaced the appreciation for tensegrity structures in behavioral and psychological sciences. The roboticists have the luxury of being able to build their own systems. The challenge for behavioral and psychological scientists is that our model-organisms’ bodies come as they are, and for centuries, we have been paying more attention to the more focal events in relatively localized, relatively stable components of our model-organisms’ anatomies. Better appreciating this tensegrity-like background, global support requires no small change in perspective, and what’s more, if the promise from both robotics and from cellular and tissue-focused biology is true, then it confounds behavioral-scientists’ best hopes to respect long-held wisdom in experimental design.

To put a finer point on it, the primacy of the randomized controlled trial (RCT) is an age-old value governing how behavioral and psychological sciences assign causal status. If the tensegrity structure is so intimately involved in so many of the better-known, more focal dynamics amongst anatomical parts, then there may be no ethical or practical control condition wherein we might remove the tensegrity structures to see what happens. Certainly, we know from recent research in regenerative medicine that one of the best supports for scaffolding the re-growth of atrophied muscle tissue is the experimental insertion of a “quilt” of extracellular matrix [33], and this evidence seems to vindicate the tensegrity hypothesis. However, if tensegrity so powerful, then removing all fascia/ECM-related tissues from entirely from a fully functioning, typically developing organism could be a debilitating treatment. So, granting tensegrity any causal status is simply not available against the benchmark of an RCT with a control or experimental condition differing from another in terms of presence/absence of the entire proposed tensegrity structure. The immediate risk of proposing hypotheses that do not submit to an RCT is that the tensegrity hypothesis may well be unfalsifiable.

An empirical-geometrical approach offers a falsifiable foothold from which behavioral and psychological scientists may develop more elaborate models of the causes. That is to say, it may be beneficial for scientists interested in the tensegrity hypothesis to focus their efforts around the proposal that tensegrity-driven systems are necessarily multifractal systems [20]. This proposal effectively collapses the complexities of multifarious types of tissues and model organisms into two facts: 1) what distinguishes tensegrity models of biological systems from tensegrity-free anatomical models is that the former exhibit nonlinear interactions across scales of space and time by definition whereas the definition of the latter relies centrally on the localization of relatively few scale-dependent structures [21] and 2) systems defined by nonlinear interactions across scales of space require multifractal modeling for exhaustive description and effective prediction whereas the latter tensegrity-free models should never behave differently from the best-fitting linear model reflecting a fundamentally Euclidean geometry of the scale-dependent parts of an anatomy [34–36]. With these two facts, it may be possible for behavioral and psychological scientists to unite their efforts despite wide disparities in model organisms (e.g., insects and humans). Hence, no matter the inaccessibility of the simplest RCT comparison of a potentially gruesome tensegrity versus tensegrity-free experimental manipulation, empirically estimating geometrical structure in organism behavior permits a falsifiable hypothesis: if tensegrity structure contributes to an observed behavior, then the empirically-estimable multifractal structure of the organism should represent that contribution. I examine phasmid postural sway from this perspective that the empirically-estimable multifractal geometry of postural sway will be reflect changes in phasmids’ ability to reduce their sway and in their tensegrity architecture’s contributions to this postural control.

### Multifractal geometry is fundamental to understanding tensegrity systems, and ultrafast response is only a symptom secondary to the multifractal-geometric mechanism

Ultimately, this paper is about elaborating tensegrity-themed perspectives and about phrasing the primacy of multifractal geometry over ultrafast responses as a privileged way forward in articulating the content of any tensegrity-based theory. The steady inventory of ultrafast responses will continue and has likely not come anywhere near to its conclusion. The crucial rhetorical value of ultrafast responses in theoretical discourse about context-sensitive, goal-directed movement coordination goes something like the following:

1. Commitments to inferential models based in neural tissue can only predict behaviors unfolding over the time that electrochemical transmission needs to unfold itself.
2. Many ultrafast responses are too fast for electrochemical transmission to precede.
3. So, what responds with ultrafast latency requires something other than electrochemical transmission.

A major payoff of accruing a large inventory of ultrafast responses is that we can add new straws to the computational-neuroscience camel’s back in the hopes that—maybe, just maybe—each latest evidence of ultrafast response will turn the scholarly tides away from computational neuroscience towards more ecological perspectives cognizant of tensegrity principles. However, a major risk of building this large inventory of ultrafast responses is that each of these ultrafast responses indicate alluringly to the “something else” other than electrochemical transmission, but this ultrafast-response rhetoric remains empty, and frankly, it seems unrealistic that effects skating upon connective tissue faster than neurons can support should alone inspire an about-face away from computational neuroscience. For instance, the fascinating work of Stepp, Moreno, and Turvey [37] showed ultrafast responses by the body in response to a lexical decision task in which participants viewed a string of letters and had to indicate with a finger movement whether the letter-string was a word or not. Crucially, torso and thigh each showed ever earlier patterns of response than the finger, even showing the same graded response to unpronounceable nonwords, pronounceable pseudowords, and real words. For those readers already skeptical that language-related behavior must be a computed unfolding of strictly neural processes (e.g., [38;39]), this finding is superb confirmation of that skepticism. However, this demonstration leaves tensegrity as “whatever the nervous system is too slow to do” and gains no deeper explanatory inroads into the issue of language-related behaviors.

In light of what we know now, simply pursuing ultrafast responses to the exclusion of using multifractal geometry is wrong-headed on many counts. We now know that the “something else” supporting ultrafast responses belongs to a class of distributed architectures called “tensegrity” because the ultrafast response involves a whole-organism integrity written into a hierarchical balance of tensions. We seem to becoming towards consensus that tensegrity architectures are best understood with multifractal geometries capable of articulating cascade-like interactions across time scales. Seeking for examples of what neurons cannot do alone seems to beget unconstructive dichotomies between tensegrity and nervous system—which dichotomies are unconstructive because the tensegrity structure of movement systems is possibly exactly what underwrites the nervous systems embedded in those movement systems. So, ultrafast responses are informative, but they bypass the more pressing question that many more scholars might want answered: namely, how the nervous system participates in the movement in non-computational but fully ecological ways.

The present work is a call to arms for ecological perspectives on movement coordination to use multifractal geometry as the lens on dexterity that Turvey and Fonseca [20] promised it could be. The days of ultrafast responses are not over, but we have geometrical tools that make explicit the nonlinear interactions across scales in movement systems. Ultrafast responses are remarkable symptoms of a system more flexible and dexterous than a computable nervous system, but they are just a downstream consequence of a distributed mechanism of hierarchical tensions. Multifractal modeling is the privileged class of observables that should lay bare the deep insights of precisely how those hierarchical tensions enact dexterous movement coordination.

### Multifractal geometry: Elaborations from and beyond linear modeling

This more specific hypothesis does demand answer to the question “What is multifractal geometry?” Certainly, on first acquaintance, the proposed greater specificity and falsifiability comes at the cost of seeming like more jargon. However, multifractality may be understood as generalization of concepts from linear statistics to encompass measured systems that do not distribute as homogeneously and independently as linear statistics assume.

#### Linear models and the autocorrelations that offer more linear variety

Linear statistics presume that systems distribute homogeneously and that each new event is independent from the previous event. Events appearing independently from the previous leads to an aggregate that has a) central tendency best approximated by the arithmetic mean and b) width beyond the central tendency best approximates by the standard deviation (*SD*). Fundamentally, the assumption of independence makes homogeneity of variability around the mean inevitable but leads specifically to the expectation that *SD* should increase with the square root of time, that is, as a single power-law of time, i.e., *SD* ~ *t*^.5^. Measured systems become less homogeneous and more patchy as those measured systems embody progressively less independence across sequential events.

The linear model does not simply fall flat and yield to multifractal modeling with the first glimmer of dependence across sequential events. After its two more famous properties of mean and *SD*, the linear model has a third and much less-heralded property, namely, the autocorrelation function. As described in the previous paragraph, perfect independence corresponds to an autocorrelation function with all of its coefficients set to zero. But as we allow our measured system to embody more dependence across its sequences of events, the autocorrelation specifies the relationship of current events to past events. It specifies this relationship as a coefficient for each of several time-lags indicating the contributions of progressively previous events. As systems grow more dependence on previous events, they exhibit autocorrelations with progressively more nonzero coefficients reflecting the effect of progressively more distant past events. So, the linear model actually has a vast capacity to model very much heterogeneity in our measured systems. Far from being novel, the foregoing points are foundational steps in linear modeling of time series for decades [40;41], but the vogue of fractal results have repeatedly blinded readers to the vast range of linear intermediary possibilities between zero-memory ordinary diffusion and truly nonlinear, long-range memory processes have, warranting repeated and renewed reminders [42–45].

The foregoing technical consideration also has implications for what evidence is necessary to motivate a full commitment to tensegrity hypotheses. In brief, a single multifractal result is consistent with but not conclusive evidence of tensegrity, but comparison to linear surrogates provides more conclusive evidence. We will have to make full use of mean, *SD*, and autocorrelation in our best-fitting linear model before we give way to multifractal modeling and before we need to admit the role of things like tensegrity structures. So, not only do we have a falsifiable null-hypothesis in needing to find deviation from the best-fitting linear model, but we should respect the breadth of parametric complexity in linear models that should stand in the way of rejecting that null hypothesis. There are very many behaviors the linear model can predict, particularly when we model the autocorrelation. It is only the greatest deviations from homogeneity and independence that should warrant espousing a multifractal geometry that is explicitly nonlinear and implicitly due to tensegrity’s promised nonlinear interactions across scales of space and time.

#### Multifractal geometry: A measure of variability for nonlinearly heterogeneous systems

Empirical estimates of multifractal geometry appear only after we introduce gradually more dependence across sequential events. We can briefly provide the short-hand understanding that multifractality is, roughly speaking, a *SD*-like measure of variability for nonlinearly heterogeneous systems. However, to give the more principled, more detailed understanding, we can see multifractality emerge from that power-law relationship of *SD*~*t*^.5^ noted above. As we build nonzero coefficients into even the shortest lags of the autocorrelation, then we will see the .5 exponent in this *SD*-defining power-law begin to move. That is, the .5 can give way to .4 or to .6 or even to .55. And the same system can embody all three as independence teeters from purity. Essentially, as independence dissolves and as tensegrity-like nonlinear interactivities appear, *SD* can follow multiple power-laws, each with different fractional exponents. And because we are now becoming concerned with tensegrity-like interactions across time as a cause, we might begin to see an importance in the variation of these fractional exponents. So, multifractality is the presence of “multiple” fractional or, for short, “fractal” exponents, and we might begin to construe multifractality as the range of these fractional exponents (i.e., maximum minus minimum). Multifractality entails that measured systems have a continuum of fractional exponents, often called a spectrum, and this range is effectively the width *W* of the multifractal spectrum.

Going one step further is to admit that *SD* is most often useful for describing variability of homogeneous processes. So, although examining the multifractality through these *SD*-defining power-laws is certainly useful [46;47], it may only be as good as the definability of the SD. As systems become more heterogeneous, *SD* may no longer be definable or may at least no longer entail what it was originally intended to for a Normal distribution, which point has been taken up in various models of animal and insect foraging (see [48] for a review). So, if we wish to take a step further away from *SD*, then it is possible as well to estimate multifractality by examining time series or spatial series data and examining how densely or sparsely the measured system distributes across various nonoverlapping bins within the measurement. Bin proportion *p* should distribute with bin size L according to a single power-law, *p*~*L*. For instance, if we take a homogeneous process and measure it across two of size *L*, ten of size .5*L*, or a hundred bins of size .01*L*, we should find the system inhabiting each of the bins the same amount of time, that is, 50% of the time in each of the *L*-size bins, 10% of the time in each of the .5*L*-size bins, and 1% of the time in each of the .01*L*-size bins. Just as the single-power-law rule for *SD* above epitomizes purely independent, purely homogeneous linear processes, that single-power-law rule of *p*~*L* epitomizes homogeneous systems. We can break it theoretically by building or simulating systems according to nonlinear interactions across scales, and in that case, as above, the range of fractional exponents on *L* indicates multifractality. I chose to deal with multifractality in this latter guise. This choice reflects two concerns. First, the latter *p*~*L* variant will not require the standard assumptions of homogeneity implicit for defining SD, and second, as noted below, the quasi-periodicity of the data makes *SD* an opaque measure that makes the traditional *SD*-based analyses of multifractality challenging.

#### Operationalizing tensegrity structure in *t*-statistics comparing multifractal-spectrum width *w* for original series to multifractal spectrum widths for best-fitting linear models

Here is a final step in operationalizing the very abstract tensegrity hypothesis. No matter the choice of multifractal analysis, there is the same benchmark of comparison to the best-fitting linear model, which will require multifractal analysis not just for the measured series but also for each of several linear-model simulations of the series that reshuffle the same values (i.e., maintaining the same mean and *SD*) in ways that preserve the same autocorrelation of the original measured series. Hence, every original measurement has a multifractal-spectrum width *W* but also a t-statistic *t*_*MF*_ that compares original *W* to the multifractal-spectrum widths resulting for each best-fitting linear-model simulations.

To bring this back to earlier concerns, our falsifiable hypotheses about the role of tensegrity structures appear here in the *t*_*MF*_. Briefly put, a significantly non-zero *t*_*MF*_ entails that there is multifractal heterogeneity beyond the bounds of what is typically expectable from the best-fitting linear model of the measured data. Furthermore, the direction of a significantly non-zero *t*_*MF*_ encodes, in very broad terms, what pattern of nonlinearity interactions across scales the tensegrity system is using.

Clarity about the *t*_*MF*_ is challenging in general and warrants further discussion especially if the *t*_*MF*_ is going to be useful for informing the tensegrity hypothesis. There is a misunderstanding in the literature that linearity entails zero multifractality (e.g., [49]). That misunderstanding begins with the sensible notion that multifractality is a useful way to quantify variability in the presence of nonlinear interactions, but it proceeds to an invalid but very tempting conclusion that there should be no multifractality in the linear case. This line of reasoning necessarily requires that the only significant *t*_*MF*_ we should find in tensegrity structures are positive. However, this line of reasoning ignores the fact that the linear autocorrelation function gives linear processes a wide capacity for minor failures of homogeneity and of independence, and these autocorrelational structures have long been known to produce spurious cases of nonzero *w* that have nothing to do with nonlinear interactions [50]. Moreover, simulation of systems with explicit nonlinear interactions across time have shown that *t*_*MF*_ becomes more often significantly negative when the interactions across time operate to counteract one another, to constrict variability, and thereby to produce less heterogeneity than the best-fitting linear model would predict [51]. Perhaps unintuitively, just as linear processes can produce more multifractal variability that some readers might expect, it is also true that linear models of the observed data can predict more than the observed data themselves.

To summarize the above, whereas a significantly nonzero *t*_*MF*_ should indicate the presence of nonlinear interactions across scales, significantly negative *t*_*MF*_ should indicate the deployment of nonlinear interactions across scales to stabilize and constrict behaviors. These two points shaped my present hypotheses about phasmid postural sway.

### A reanalysis of postural-displacement series in phasmids

The present work was a reanalysis of postural-displacement series of phasmids perched on a dowel who experience, in one condition, wind stimulation from a household fan and, in another condition, no wind stimulation. The original work documented postural sway in phasmids and suggested that it was an evolutionary adaptation to support motion camouflage, allowing phasmids to blend in with their surroundings. However, the same research found that, without plants to camouflage the phasmid, the phasmid postural sway decreased with continued wind stimulation. That is to say, wind increased phasmids’ postural sway in the short term but decreased it in the longer-term term. Hence, how ever natural and comfortable it may be for phasmids to perch upon a branch in windy conditions, these phasmids experienced more sway when the wind began, and they acted to constrain their sway immediately after the wind began. Hence, the explanation of sway as an evolutionary adaptation to partake in motion camouflage fell short, and there is some amount of the change in sway not yet explained. I investigated the possibility that the phasmid postural sway exhibited multifractal structure and, furthermore, the possibility that empirical estimates of multifractality in postural sway could reveal the tensegrity-based contributions to reducing sway.

I applied multifractal modeling to phasmid postural sway with two specific goals. The first goal was to showcase multifractal spectrum width *W* as a variability measure appropriate for heterogeneous measured systems, but the second goal was to demonstrate a significantly non-zero *t*_*MF*_ with specifically negative sign indicating the sway-reducing response that wind stimulation provokes. As for the first goal, the multifractal-spectrum width *W* should likely exhibit the same pattern that as variability measures for postural sway did in the original report on these data: an initial increase due to wind stimulation and a smooth decrease as the phasmids acclimate to the wind stimulation. Indeed, prior work examining posture has shown that greater sway corresponds to greater multifractality [3,4]. It may hopefully only serve to confirm the intuitive similarities between multifractal spectrum width *w* and *SD*. The second goal was an aim to determine whether postural-sway dynamics in phasmids reflected the generic principles of the tensegrity hypothesis, and behind this aim was a proposal that the *t*_*MF*_ should speak to the perceptual-motor response to the initially sway-increasing wind stimulation. After the phasmids had withstood an initial period of wind stimulation and heightened sway, the sway-reducing action of the tensegrity structure under these conditions might manifest as a significant decrease in *t*_*MF*_ over the period just following the initially sway-increasing stimulus.

#### “Block” versus “all”-series multifractal estimates *W* and *t*

The present hypotheses had to be clear with regards to the time scales available in the present dataset. The original report on these data included data collection over an entire two minute span in each condition, and the original report focused on postural sway in the initial 20 seconds of stimulation. I aimed to analyze the change over four 20-s blocks as well as in the context of the longer behaviors. There were only four because, as the original authors’ documentation of their dataset indicate, wind stimulation did not begin uniformly at the beginning of the 2 minute video recording used to record postural sway. Past four blocks, the number of phasmids with a fifth full 20-second block decreased drastically, ensuring that a regression model of 5 20-s blocks would have been extremely unbalanced. Specifically, the present hypotheses focused explicitly on these 20-s and so the present results focused on the multifractal indicators *W* and *t*_*MF*_ for individual blocks. But it was expected that each insects’ responses through these variables *W* and *t*_*MF*_ for individual blocks should depend on the same insects’ multifractal structure *W* and *t*_*MF*_ across the entire 2-minute series with stimulation and the entire 2-minute series without stimulation.

## Two hypotheses

Hence, to further clarify the hypotheses, it is important to note that they were expectations that *W*_*BLOCK*_ and *t*_*BLOCK*_ (i.e., the multifractal spectrum width *W* for a 20-second block and t-statistic *t*_*MF*_ comparing original *w* to linear models for a 20-second block) to change in the aforementioned patterns but moderated by *w*_*ALL*_ and *t*_*ALL*_ (i.e., the multifractal spectrum width *W* for all 2 minutes and t-statistic *t*_*MF*_ comparing original *W* to linear models for all 2 minutes), respectively. In this more subtle phrasing, the first hypothesis was that *W*_*BLOCK*_ would initially increase for phasmids under wind stimulation but decrease smoothly across blocks with this decrease moderated by *W*_*ALL*_. The second hypothesis was that *t*_*BLOCK*_ would decrease abruptly on the second block, with the decrease moderated by *t*_*ALL*_ and, once the tensegrity structure had served the purpose of reducing the phasmid’s postural sway, not again. Both results supported both hypotheses.

## Materials and methods

A repeated-measures design examined postural sway in each of 21 phasmids standing on a 9mm-diameter dowel suspended in a hollow frame (20×30×26 cm^2^) under two conditions in randomly counterbalanced order. In one condition, a Sunbeam household pedestal fan positioned to one side of the phasmid produced a wind-like stimulus, approximating wind speed of 2m/s at the phasmid’s perch. In the other condition, the fan was off.

Two Panasonic HDC-SD80 Full HD Camcorder video cameras positioned to have perpendicular views of the phasmid recorded two 2-dimensional images for subsequent combination into 3-dimensional position data from a point on the phasmid’s abdomen. A postural-displacement series is defined as the series of Euclidean distances between consecutive pairs of points. Data are from the original study [1] whose authors may be contacted at Richard.Peters@latrobe.edu.au. A minimal dataset used for this specific study and reanalysis is available for download through Dryad Digital Repository (https://dx.doi.org/10.5061/dryad.v52dp25).

### Data analysis

#### Multifractal analysis

I sought to model the local changes in multifractal structure of 1000-frame blocks of the postural-displacement series. That is, one model addressed the dependent variable of *W*_*BLOCK*_, multifractal spectrum width by block, in terms of predictors including the presence/absence of wind stimulation and the entire postural-displacement series *W*_*ALL*_. Another model addressed the dependent variable of *t*_*BLOCK*_, multifractal-spectrum width due to nonlinearity by block, in terms of predictors including the presence/absence of wind stimulation and the entire postural-displacement series *t*_*ALL*_. This section reviews the algorithms for estimating *W* and *t*.

Chhabra and Jensen’s [52] canonical “direct” algorithm allowed estimating multifractal-spectrum width *W* by sampling measurement series *u*(*t*) at progressively larger scales. Proportion *P*_*i*_(*L*) within bin *i* of scale *L* is

$$P_{i}\left(L\right)=\frac{\sum_{k=\left(i-1\right)L+1}^{iL}u\left(k\right)}{\sum u\left(t\right)}$$ (1)

CJ method using parameter *q* to convert *P*(*L*) for *N*_*L*_ nonoverlapping *L*-sized bins of *u*(*t*) and generate mass *μ*_*i*_(*q*,*L*):

$$\mu_{ij}(q,L_{j})=\frac{{\left[P_{ij}(L_{j})\right]}^{q}}{\sum_{i=1}^{N_{j}}{\left[P_{ij}(L_{j})\right]}^{q}}.$$ (2)

For each *q*, each estimated *α*(*q*) appears in the multifractal spectrum only when Shannon entropy of *μ*(*q*,*L*) scales with *L* according to the Hausdorff dimension *f* (*q*),where

$$\begin{array}{l} f(\alpha (q))=-\underset{N_{j}\to \infty }{lim}\frac{\sum_{i=1}^{N_{j}}\mu_{ij}(q,L_{j})ln[\mu_{ij}(q,L_{j})]}{lnN_{j}}\\ f(\alpha (q))=\underset{L_{j}\to 0}{lim}\frac{\sum_{i=1}^{N_{j}}\mu_{ij}(q,L_{j})ln[\mu_{ij}(q,L_{j})]}{lnL_{j}} \end{array}$$ (3)

and where

$$\begin{array}{l} \alpha (q)=-\underset{N_{j}\to \infty }{lim}\frac{\sum_{i=1}^{N_{j}}\mu_{ij}(q,L_{j})ln[P_{ij}(L_{j})]}{lnN_{j}}\\ \alpha (q)=\underset{L_{j}\to 0}{lim}\frac{\sum_{i=1}^{N_{j}}\mu_{ij}(q,L_{j})ln[P_{ij}(L_{j})]}{lnL_{j}}. \end{array}$$ (4)

The scaling region used for estimates of *W*_*ALL*_ was 4 samples to 1503 samples for the entire 6012-sample postural-displacement series. The scaling region used for estimates of *W*_*BLOCK*_ was 4 samples to 250 samples for the 1000-sample subsets of the entire postural-displacement series. For -200≤*q*≤200, and including only linear relationships with correlation coefficient *r*>.995 for Eqs 3 and 4, the downward-opening curve **(***α*(*q*),*f*(*q*)**)** is the multifractal spectrum. *α*_*max*_-*α*_*min*_ is multifractal-spectrum width *W* according to the CJ algorithm. I used the CJ algorithm because the postural-displacement series exhibited quasiperiodicities, a known challenge to popular finite-variance scaling methods [53].

The range of *q* available to test may seem alarming, but it follows in the tradition of theoretical recommendations from Mandelbrot [54] and empirical recommendations from Zamir [55]. Essentially, both scholars recognized that binomial cascades are a poor standard for parametrization of *q* for multifractal analysis on empirical data, particularly for multifractal algorithms resorting to Legendre transformations of the partition function. Both scholars addressed the CJ algorithm by name as an alternative to this Legendre-transformation-of-partition-function type and so as an avenue for testing more *q* than otherwise.

**Fig 1:**
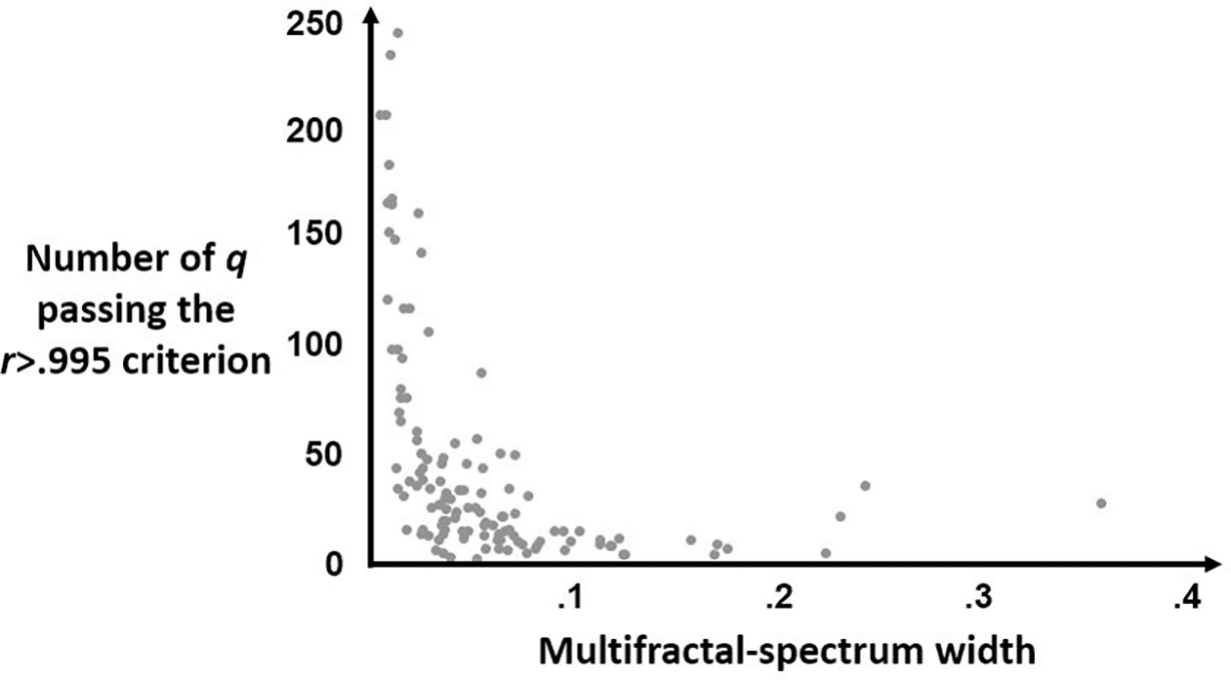
Exponents *q* for which multifractal analysis met the *r* < .995 benchmark for Eqs 3 and 4. Plot of individual series’ multifractal analysis indicating, on the y-axis, how many values of *q* served to produce stable power-law relationships in Eqs 3 and 4 and, on the x-axis, the resulting width of the multifractal spectrum. Because *q* is effectively a distortion serving to reveal differences in the temporal structure, less multifractal series should withstand a wider range of *q* and generate more similar and all equally stable scaling relationships in Eqs 3 and 4. However, more multifractal series will have more heterogeneous structure that will be more likely to generate deviations in and eventually weaknesses in power-law relationships with smaller changes in *q*.

As Zamir [55] noted, physiological data has structure that interacts with multifractal analysis to destabilize the power-law forms required for proper estimation. The exponent *q* is effectively a parameter that distorts a measured series in order to estimate potentially different temporal structure for fluctuations of different sizes. The least multifractal data will have the most homogeneous distribution of fluctuation sizes and, more crucially, the most homogeneous temporal structure across all different fluctuation sizes. Hence, variation in *q* should have no effect on the temporal structure, entailing that very many *q* should leave the very same power-law scaling in temporal structure. The most multifractal data should, conversely, show the most difference in power-law scaling in the fewest number of *q*. In short, the perturbation entailed by *q* should make the least change for data with little to no multifractality and show the greatest stability across a very wide range of *q*. There should, on the other hand, be a more rapid decay in the fidelity of power-law scaling (i.e., in the *r* expressing the relationship between numerators and denominators in Eqs 3 and 4) for more multifractal data as we increase the bounds of *q*. We offer Fig 1 as an example of how, in this data set, the number of acceptable *q* according to the *r*>.995 standard was inversely proportional to the width of the multifractal spectrum.

The briefer point that Fig 1 may illustrate is that this policy of testing a wide range of does not lead to spurious widenings of the estimated multifractal spectrum. On the contrary, more values of *q* serve only to solidify evidence of less multifractality.

**Fig 2:**
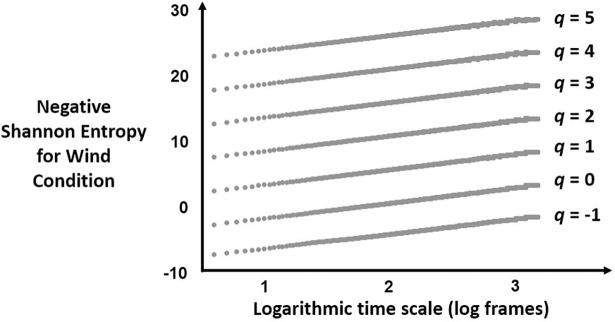
Scaling relationships for Eq 3 for the Wind condition. Plot of the negative Shannon entropy on y-axis against logarithmic time scale for an example postural-displacement series in the Wind condition for each of 7 values of *q*.

**Fig 3:**
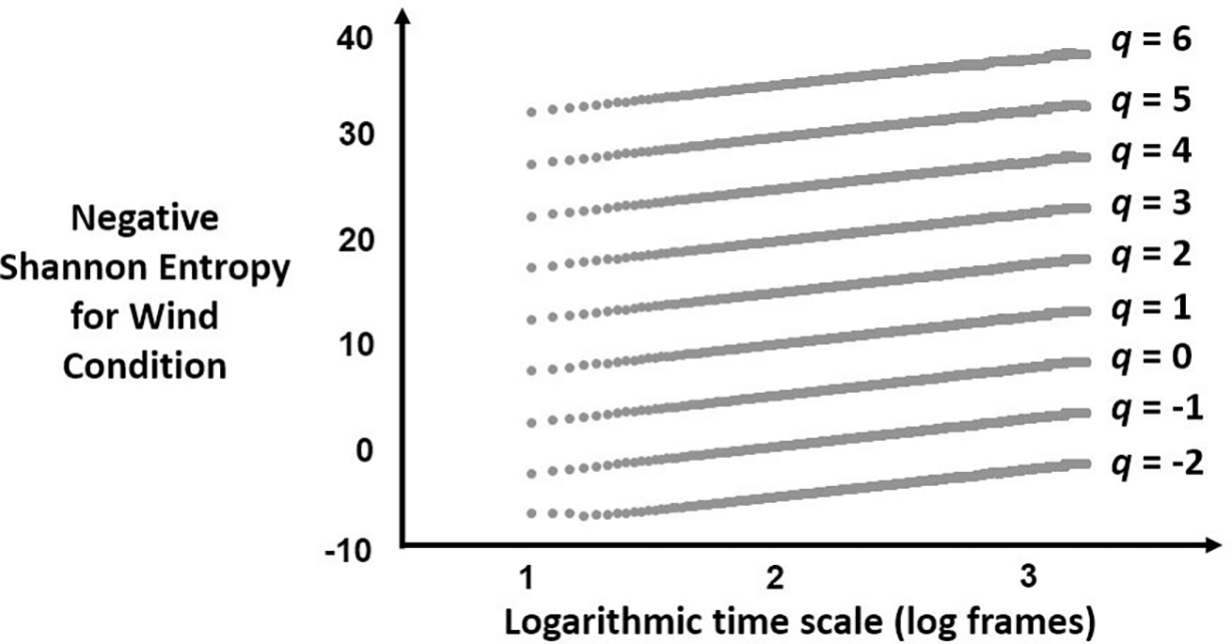
Scaling relationships for Eq 3 for the No-Wind condition. Plot of the negative Shannon entropy on y-axis against logarithmic time scale for an example postural-displacement series in the No-Wind condition for each of 9 values of *q*.

Figs 2 and 3 depict the relationship in Eq 3 between time scale and negative Shannon entropy for an example series in the Wind and the No-Wind conditions, respectively.

#### Calculating t from comparison to Iterated Amplitude Adjusted Fourier-Transform (IAAFT) surrogates

50 IAAFT surrogates [56] were produced for each original inter-reading interval series, using 1000 iterations of randomizing the phase spectrum from the Fourier transform, taking the inverse Fourier transform of the original series’ amplitude spectrum with the randomized phase spectrum, and replacing in the inverse-Fourier series with rank-matched values of the original series. We calculated *t* as the difference $\left(W-(\frac{1}{50})\sum_{i=1}^{50}W_{Surr}(i)\right)$ divided by the standard error of *W*_*Surr*_. Hence, positive or negative *t* indicated wider or narrower, respectively, spectra than surrogates.

#### Mixed-effect linear modeling

My aim was to model change in repeated measures for specific organisms over time. The ideal framework for doing so is mixed-effect linear modeling [57]. Repeated-measures ANOVA is capable of fitting random-effect individual-participants intercepts to control for unsystematic inter-individual differences, but it falls short in the sense that any ANOVA factor of time (e.g., Block) is only a categorical distinction amongst different values. There is no appropriate way in ANOVA to encode the directionality of time, and there is likewise an assumption in standard ANOVA frameworks that covariates (e.g., *W*_*ALL*_ or *t*_*ALL*_) should not have significant interactions with fixed effects. On the other hand, mixed-effect linear modeling allows a framework for testing change over time with random-effect intercepts, directionality in time effects, and allows for judicious estimation of interactions between covariates and fixed effects.

The function “lmer” in the R package “lme4” allowed linear mixed-effect modeling [58] as well as R package “lmerTest” for the Satterthwaite estimation of *F*-test-based *p*-values for fixed effects [59]. Predictors included a random-effects intercept for each phasmid the following fixed effects: Condition (1 = wind, 0 = non-wind), Block (block number 1, 2, 3, and 4), as well as *W*_*ALL*_ and *t*_*ALL*_ (the multifractal-spectrum width and the multifractality due to nonlinearity, respectively, of the entire displacement series). As noted above, *W*_*ALL*_ and *t*_*ALL*_ each appear only in the model of the corresponding by-block measure, i.e., *W*_*BLOCK*_ and *t*_*BLOCK*_. One model uses orthogonal polynomials using R’s “poly” function to estimate smooth change with Block, and the other model uses R’s “as.factor” function to fit the general-representation of time in which Block values 2, 3, and 4 each receive their own intercepts to encode their differences from Block 1 (e.g., [57]).

## Results and discussion

### Entire postural-displacement series: Similar multifractal-spectrum width, disparate modes of deviating from the linear surrogates

For the condition receiving wind stimulation (Condition = 1), the entire postural-displacement series had all-signal multifractal-spectrum width *W*_*ALL*_ averaging .0757 (*SD* = .0529), all-signal multifractal-spectrum width due to nonlinearity *t*_*ALL*_ averaging -4.5850 (*SD* = 14.2596), with 7 and 11 of the 21 signals having *t*_*ALL*_ greater than 1.96 and lesser than -1.96, respectively.

For the condition not receiving wind stimulation (Condition = 0), the entire postural-displacement series had all-signal multifractal-spectrum width *W*_*ALL*_ averaging .0622 (*SD* = .0458), all-signal multifractal-spectrum width due to nonlinearity *t*_*ALL*_ averaging 6.044 (*SD* = 17.2953), with 10 and 9 of the 21 signals having *t*_*ALL*_ greater than 1.96 and lesser than -1.96, respectively.

Hence, the two conditions yielded postural displacements with comparable multifractal-spectrum width, but that similarity belies the disparity in how these conditions evoked nonlinearity. When compared to each corresponding linear surrogate, the postural-displacement series had significantly narrower multifractal spectra than their surrogates did, and the postural-displacement series had significantly wider multifractal spectra than their surrogates did. Thus, postural sway in phasmids exhibits nonlinearity deviating from the time-symmetry of a linear process, but whereas postural sway without wind stimulation exhibits that sort of nonlinearity in which interactions across time series make postural displacements more variable than linearly expectable, the wind stimulation elicited nonlinear patterns in which the interactions across time scale served to constrict variability below that typically expected for linear processes.

### Effects on block-by-block multifractal-spectrum width *W*_*BLOCK*_

**Fig 4:**
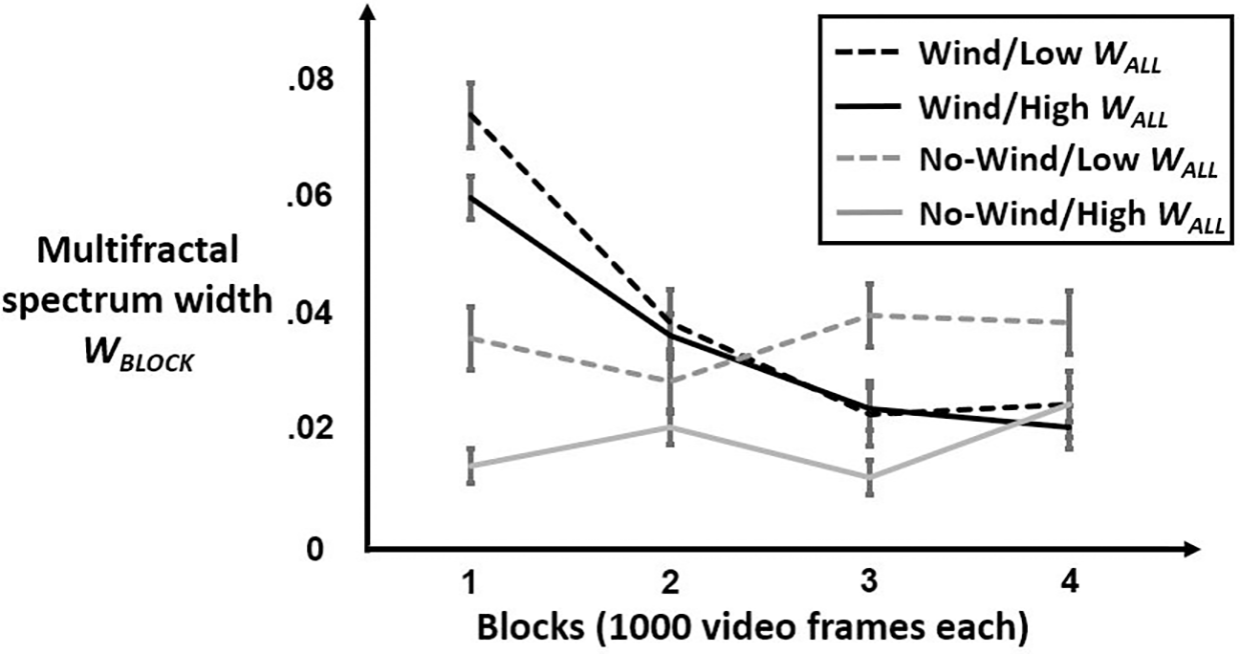
Model predictions for *W*_*BLOCK*_. Plot of model predicted *W*_*BLOCK*_ over four 20-s (1000-frame) blocks for phasmids with high *W*_*ALL*_ (solid lines) and with low *W*_*ALL*_ (dashed lines) under the conditions of wind stimulation (black lines) or no-wind stimulation (grey lines). In all cases, “high” and “low” were defined as the third and first quartiles in corresponding conditions. *W*_*BLOCK*_ was initially much greater at the onset of wind stimulation, but it decreased smoothly across blocks, showing a quicker decrease for phasmids who had lower multifractal spectrum width across their whole series. Phasmids in the no-wind condition showed initially much lower *W*_*BLOCK*_ and only very shallow increase of *W*_*BLOCK*_ with progressive blocks.

A regression model of multifractal-spectrum width by block *W*_*BLOCK*_ returned significant effects of Condition; W_ALL_; the interaction Condition×W_ALL_; the interaction of Condition with orthogonal linear, quadratic, and cubic polynomials of Block; as well as the interactions of Condition, W_ALL_, and Condition×W_ALL_ each with the orthogonal cubic polynomial of Block. Fig 4 shows the model predictions this model, and the next paragraph outlines the specific effects contributing to these model predictions.

Table 1 reports coefficients from a regression model of *W*_*BLOCK*_. *W*_*BLOCK*_ was higher for the Condition receiving wind stimulation (*B* = .0446, *SE* = .0062, *p* < .0001) and for phasmids with higher W_ALL_ across their entire postural displacements (*B* = .5288, *SE* = .0618, *p* < .0001), but it was lower for phasmids with higher W_ALL_ in the Condition (*B* = -.6129, *SE* = .0786, *p* < .0001). W_ALL_ moderated the cubic decrease of W_BLOCK_ across blocks for all phasmids (*B* = -2.9108, *SE* = .6520, *p* < .001). Although Condition counteracted this W_ALL_xBlock(Cubic) effect (*B* = 2.9180, *SE* = .8504, *p* < .001), Condition made its own independent contribution to the cubic decrease (*B* = -.1539, *SE* = .0704, *p* < .05) as well as producing a negative linear decrease (*B* = -.3371, *SE* = .0743, *p* < .0001) made steeper with a positive quadratic (*B* = .1478, *SE* = .0711, *p* < .05) to a local minimum. The positive quadratic entails an increase over the last two blocks just visible in the low-*W*_*ALL*_ case but overwhelmed by the significant negative cubic effects in the high-*W*_*ALL*_ case.

**Table 1:** Regression model predicting the changes in *W*_*BLOCK*_, multifractal spectrum width for consecutive 1000-frame portions of the postural displacements.

Predictor	*B*	*SE*	*p*
Intercept	.0031	.0052	.55
Condition	.0446	.0062	< .0001
Block(Linear)	.0407	.0570	.47
Block(Quadratic)	.0195	.0520	.70
Block(Cubic)	.1498	.0501	< .01
*W*_*ALL*_	.5288	.0618	< .0001
Condition×Block(Linear)	-.3371	.0743	< .0001
Condition×Block(Quadratic)	.1478	.0711	< .05
Condition×Block(Cubic)	-.1539	.0703	< .05
Condition×*W*_*ALL*_	-.6129	.0786	< .0001
Block(Linear)×*W*_*ALL*_	-.1289	.7136	.85
Block(Quadratic)×*W*_*ALL*_	.0460	.6645	.94
Block(Cubic)×*W*_*ALL*_	-2.9108	.6520	< .001
Condition×Block(Linear)×*W*_*ALL*_	.9805	.9191	.29
Condition×Block(Quadratic)×*W*_*ALL*_	-.9646	.8633	.27
Condition×Block(Cubic)×*W*_*ALL*_	2.9180	.8504	< .001

### Effects on block-by-block multifractal-spectrum width due to nonlinearity *t*_*BLOCK*_

**Fig 5:**
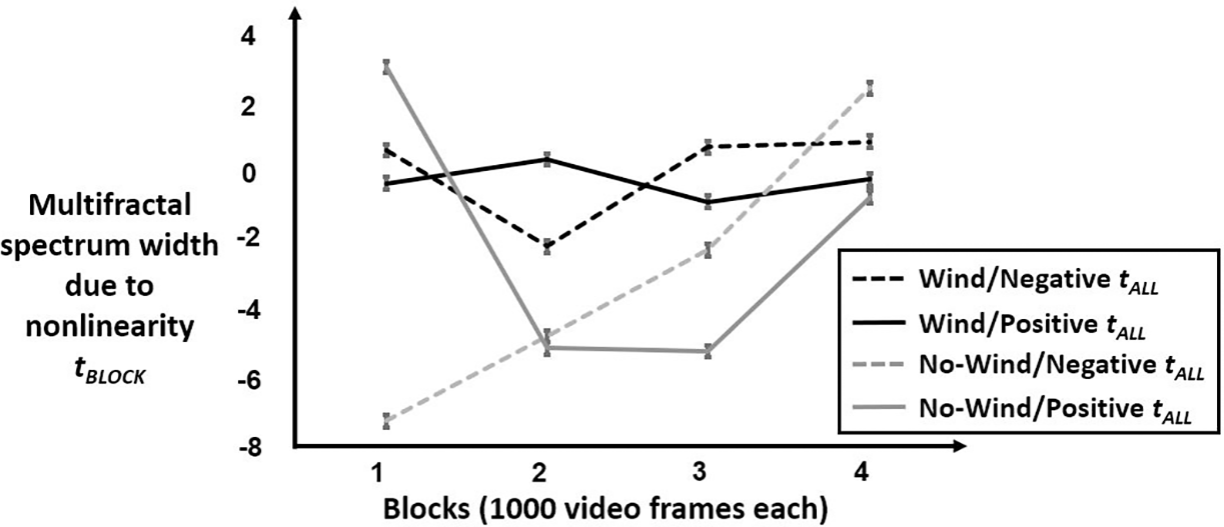
Model predictions for *t*_*BLOCK*_. Plot of model predicted *t*_*BLOCK*_ over four 20-s (1000-frame blocks for phasmids with positive *t*_*ALL*_ (solid lines) and with negative *t*_*ALL*_ (dashed lines) under the conditions of wind stimulation (black lines) or no-wind stimulation (grey lines). In all cases, “positive” and “negative” settings of *t*_*ALL*_ for these plots were defined as the third and first quartiles of *t*_*ALL*_ in corresponding conditions. *t*_*BLOCK*_ was effectively flat across all blocks of wind stimulation, with the exception of a significant decrease (e.g., to -2.10 for the third quartile *t*_*ALL*_ in wind stimulation). Phasmids in the no-wind condition showed *t*_*BLOCK*_ comparable to their *t*_*ALL*_ in the first block. Subsequent blocks without wind stimulation showed negative *t*_*BLOCK*_ for all cases on Blocks 2 and 3, increasing on Block 4.

An important difference from the model for *W*_*BLOCK*_ is that there were no polynomial effects, and rather, the model for *t*_*BLOCK*_ returned significant effects of Block only for Block coded as a class variable, that is, treating time in generic terms assigning each value of Block its own intercept shift, with Block 1 treated as the comparison case for which the regression [57]. Fig 5 shows the model predictions this model, and the next paragraph outlines the specific effects contributing to these model predictions.

Table 2 reports coefficients from a regression model of *t*_*BLOCK*_. A significant negative intercept (*B* = -4.7088, *SE* = 2.2781, *p* < .05) reflected the average tendency towards negative values of *t*_*BLOCK*_. All values of *t*_*BLOCK*_ were significantly higher on Block 4 across conditions (*B* = 6.5037, *SE* = 3.2635, *p* < .05) suggesting that sustained time acclimating led eventually but discontinuously to reversals of the initially negative direction of *t*_*BLOCK*_. Phasmids with higher *t*_*ALL*_ across their entire postural displacements had higher *t*_*BLOCK*_ (*B* = .3843, *SE* = .1272, *p* < .01) in the first Block in the absence of wind stimulation, but the Condition with wind stimulation led phasmids with higher *t*_*ALL*_ to exhibit relatively more negative *t*_*BLOCK*_ (*B* = -.4420, *SE* = .1970, *p* < .05) in that first Block. After Block 1, phasmids with higher *t*_*ALL*_ had more negative *t*_*BLOCK*_ in Block 2, 3, and 4 (*B*s = -.3975, -.4943, and -.5028, respectively; *SE*s = .1799, .1799, and .1800, respectively; *p*s < .05, .01, and .01, respectively), all significantly lower than that for Block 1 but none of which were significantly from one another among Blocks 2 through 4.

**Table 2:** Regression model predicting the changes in *t*_*BLOCK*_, multifractal spectrum width due to nonlinearity for consecutive 1000-frame portions of the postural displacements.

Predictor	*B*	*SE*	*P*
Intercept	-4.7088	2.2781	< .05
Condition	4.7452	3.1693	.14
Block2	-.1122	3.2217	.97
Block3	1.79	3.2217	.57
Block4	6.5037	3.2635	< .05
*t*_*ALL*_	.3843	.1272	< .01
Condition×Block2	-.184	4.4821	.96
Condition×Block3	2.1567	4.4821	.63
Condition×Block4	6.3514	4.5347	.16
Condition×*t*_*ALL*_	-.442	.197	< .05
Block2×*t*_*ALL*_	-.3975	.1799	< .05
Block3×*t*_*ALL*_	-.4943	.1799	< .01
Block4×*t*_*ALL*_	-.5028	.18	< .01
Condition×Block2×*t*_*ALL*_	.6082	.2786	< .05
Condition×Block3×*t*_*ALL*_	.4562	.2786	.10
Condition×Block4×*t*_*ALL*_	.4943	.2786	.08

Phasmid postural-displacement showed a response specific to wind-stimulation on Block 2 and on no further blocks afterwards. Specifically, for the Condition just having received wind stimulation in Block 1, Block-2 *t*_*BLOCK*_ was moderated by *t*_*ALL*_ (*B* = .6082, *SE* = .2786, *p* < .001). Given that the Condition receiving wind stimulation had relatively more negative *t*_*ALL*_, this effect indicates that postural-displacements manifested greater nonlinearity in the specific direction of narrowing multifractal-spectrum width compared to multifractality expected from linear surrogates.

### Conclusion

The present reanalysis of data from [1] tested two hypotheses. The first hypothesis was that wind stimulation would initially increase multifractal spectrum width over the first 20-second block *W*_*BLOCK*_ and that continued experience acclimating to wind stimulation would lead to a smoothly decreasing multifractal-spectrum width over the subsequent 20-s blocks, with the decrease moderated by the entire series multifractal spectrum width *W*_*ALL*_. The second hypothesis was that *t*_*ALL*_ would be significantly non-zero in all cases and relatively more negative with wind stimulation than in no-wind condition and that the second 20-s block would exhibit a significantly negative *t*_*BLOCK*_ under wind stimulation and moderated by *t*_*ALL*_, indicating the tensegrity-like nonlinear interactions across scale acting to stabilize posture by constricting sway. Results supported both hypotheses.

The results for the first hypothesis confirm the intuition that multifractal-spectrum width has an intuitive relationship to postural sway (e.g., [3–5]). Results for the second hypothesis indicate that the phasmid postural sway has the geometrical properties expected from the tensegrity hypothesis and that the tensegrity supporting postural sway uses its nonlinear interactions across scale to constrict variability and reduce sway under the initially sway-increasing wind stimulation.

#### Tensegrity-based “stability” is not “less sway,” but it can explain reductions of sway when motion camouflage cannot

An important caveat here is that the appearance of “tensegrity” as a sway-reducing mechanism in the present reanalysis is by no means the general rule. And this caveat contains within it lessons about ecologically valid definitions of stability based on movement-based outcomes. Bian et al. [1] approached postural sway as an evolutionary adaptation that might contribute to motion camouflage allowing the phasmid to blend in with foliage waving in the breeze. Hence, just as human movement science is ready to acknowledge that the healthy outcome “continued upright postural stance” depends on healthy amounts of sway [15–19], Bian et al. understood that sway could be a life-preserving feature of phasmid posture. Sway in the short-term might seem to be a lack of stability, but in the longer-term of going unnoticed by a predator, sway becomes a guarantee of stability. The problem for this evolutionary-adaptation explanation was that sway-producing wind stimulation ended up prompting a *reduction* in sway. Hence, no matter the sensible movement-outcome focus of Bian et al.’s [1] view of postural sway, they failed to find the evidence that phasmids exploited the wind in the way they predicted. If phasmids would need to sway more to engage in motion camouflage, the reduction of sway appeared either like a morbid wish to stand out to predators or more straightforwardly like a failure of this particular adaptationist account of sway.

It was in this shortfall in the evolutionary survival account where I saw an opportunity to test the tensegrity hypothesis: multifractal structure might reveal an explanation for the reduction in sway. Explanatorily, an evolutionary imperative to survive through motion camouflage and so to avoid predators will lead wind stimulation to prompt more sway in the long term. However, the facts of postural sway are much more nonlinear than this evolutionary account predicts. Indeed, the finding that inspired Bian et al.’s work was Bässler and Pflüger’s [60] observation that phasmids generating postural sway in response to extremely subtle perturbations in the ground surface. So, subtle perturbations can prompt surprisingly large sway response, but then Bian et al. found that large perturbations can prompt a restriction of sway. I regret not to have had access to Bassler and Pflueger’s data, but the latter case of sway-producing stimuli leading to restricted sway seems the more challenging phenomenon to account for. I suspect that multifractal aspects of sway could as well predict the large sway response to subtle perturbations of the ground surface, but tensegrity hypotheses and their implication of multifractal geometry may have marginally better success explaining those aspects of sway that do not fall yet within the scope of evolutionary accounts.

Ultimately, human movement science needs ecologically valid definitions of stability because “stability” is not just “less sway.” However, an important challenge of current movement science is that, despite recognizing that “some sway” is better than “no sway,” despite recognizing that variability can be an important substrate for supporting movement coordination [61], there is currently no straightforward verdict on what amount or type of sway is “good” for stability. The evidence recommending building variability into training regimens seems fraught with failures to replicate [62] and findings of strong inter-individual differences [63]. Intuitions about what should be adaptive seem muddled, and it may be precisely such a muddle that multifractal modeling could rescue us from [64]. The capacity for multifractal modeling for finding differences in sway according to pathology suggests a usefulness in detecting “bad sway” [65;66], but I am advocating here for the use of multifractal modeling even within the healthy cases. At the very least, multifractal modeling may provide a theoretical language with which to examine the tensegrity structure until we gain better intuitions about what it might be that evolutionary adaptation serves. I am not suggesting that tensegrity has not evolved like all other biological structures, but I am suggesting that, until we understand that evolution and the values governing the evolution of tensegrity structures, multifractal modeling offers a way to understand tensegrity structures without needing to know that evolutionary process completely.

#### Multifractal modeling may support movement science across species and beyond the rhetorical value of ultrafast responses

The importance of this work is that it offers a new lens through which to find similarities in movement coordination across radically disparate species. No matter the disparity in anatomical configuration, humans and phasmids appear both to sway with multifractal complexity, and both species’ postural sway has multifractal complexity significantly different from the best-fitting linear model of sway. Postural sway may follow from similar tensegrity-like principles across different species, and it may be that *t*_*MF*_ statistics offer a way to classify the type of nonlinear interactions across scale that the tensegrity systems can use to support context-sensitive behavior.

As for helping to elaborate the tensegrity hypothesis, the usefulness of multifractal modeling may bring further benefits. The foregoing work has proposed that multifractal modeling in conjunction with comparison of multifractal results with best-fitting linear models provides a falsifiable hypothesis. If multifractal modeling can provide a falsifiable window onto tensegrity principles supporting context-sensitive behavior, then it may provide a common language with which the empirical research on various model organisms can integrate a common understanding. An important promise of the tensegrity hypothesis lies in the possibility that tensegrity principles span multiple scales, and a corollary of that promise may be that the tensegrity hypothesis should apply invariantly across organisms whose morphology and behavior reside within nonoverlapping scales.

The potential efficacy of multifractal modeling for quantifying the contributions of tensegrity principles to observed behaviors could also refine the tensegrity hypothesis beyond the bare demonstration of ultrafast responses. No doubt, ultrafast responses present an elegant contrast in which it becomes clear that organismic behavior is faster and more context-sensitive than neural dynamics are able to support. However, the future of tensegrity hypotheses for behavioral and psychological sciences may lie ahead in the challenge of articulating the coordinations of tensegrity structures in conjunction with neural dynamics and not simply before neural dynamics. That is to say, a tensegrity hypothesis that focuses only on the ultrafast response may be missing out on the rich possibilities of modeling how tensegrity could have an ongoing contribution to neural events long after the ultrafast response and even after the neural events have begun. The current state of the art is for the estimation of multifractal geometry to answer questions about the relevance of cascade-like tensegrity structures. Simply stopping short at ultrafast responses is to cheapen the full form of the modern tensegrity hypothesis, and multifractal modeling may now be the proper, privileged way to convert the tantalizing ideas behind tensegrity-themed theories into empirical answers to our questions about movement coordination.
